# No Family Clustering in Behçet’s Syndrome

**DOI:** 10.4274/balkanmedj.galenos.2018.2018.1723

**Published:** 2019-02-28

**Authors:** Mehmet Engin Tezcan

**Affiliations:** 1 Clinic of Rheumatology, İstanbul Kartal Dr. Lütfi Kırdar Training and Research Hospital, İstanbul, Turkey

To the Editor,

Behçet’s syndrome is considered as a syndrome because of its diverse clinical expressions. Patients with Behçet disease have been subclassified into various clusters on the basis of clinical expressions (1). The reason for the existence of different types of clusters in Behçet syndrome is currently unknown.

Similar to that of most diseases, genetic and environmental factors participate in the pathogenesis of Behçet syndrome. The exact contribution of these factors to the emergence of Behçet disease, however, is unknown. Therefore, I conducted a basic study that is based on a simple questionnaire to examine the hypothesis *“Behçet disease* *patients in the same family generally accumulate in similar clinical clusters”*. The questionnaire used in this study had been previously evaluated in a large cohort (2). However, I wanted to reassess the hypothesis in my Behçet disease cohort.

Given that patients with Behçet syndrome have heterogeneous clinical expressions and universal diagnostic criteria for Behçet disease do not exist, I used the International Study Group Criteria for Behçet Disease criteria to standardize the study group (3). Thus, all patients in the study fulfilled International Study Group Criteria for Behçet Disease criteria. I also clinically subclassified the patients to one Behçet’s syndrome cluster. As previously described, these Behçet disease clusters included the mucocutaneous, vascular, enthesis–arthritis–acne, and eye disease clusters (1). Additionally, I classified patients into neurologic, intestinal, and undetermined clusters for clinical purposes. This study was approved by the local research ethics committee and performed in compliance with the Helsinki Declaration.

Eighty-five patients and their relatives provided consent to participate in this study. First, I re-evaluated the family histories of patients with Behçet disease. Then, I asked patients the question *“Do you have any first- or second-degree relatives with one *Behçet disease*-related symptoms, including oral and genital ulcers, eye disease, cerebral events, skin lesions, vascular thrombosis, hemoptysis, or pulmonary symptoms?”* Lastly, I interviewed the relatives of the patients that replied to the latter question in the affirmative or have been already diagnosed with Behçet disease. I checked their symptoms against International Study Group Criteria for Behçet Disease criteria and subclassified participants who fulfilled International Study Group Criteria for Behçet Disease criteria to a Behçet disease cluster.

The patients were classified most frequently to the mucocutaneous cluster and then to the eye cluster. Furthermore, 22 (25.9%) of the patients had first- or second-degree relatives with Behçet disease. The enthesis–arthritis–acne cluster had the highest frequency of family history. No apparent accumulation of similar findings in index Behçet disease cases and their close relatives was observed (Table 1). Karaca et al. (2) previously showed that only the papulopustular and arthritis cluster may show familial clustering.

The results of these studies did not provide support for my first hypothesis that *“Behçet disease patients in the same family generally accumulate in similar clinical clusters”*. Nevertheless, genetics may have a major role in the emergence of Behçet disease given that it is accepted as a member of a group of diseases called major histocompatibility complex-1 opathy (4). Furthermore, on an epigenetic level, DNA methylation in several gene loci is related to Behçet disease. Similar to that of most diseases, genetic background (e.g., alterations in major histocompatibility complex-1 related genes) is insufficient for the full-blown expression of Behçet disease. I have a second hypothesis that even against a similar genetic background, multiple and separate hits of environmental or non-genetic factors may participate in the pathogenesis of Behçet disease. Environmental factors may also alter DNA methylation. The signs of Behçet disease are expressed individually at a time during ensuing relapses. For example, in the vascular cluster, different types of vascular involvements usually manifest at each successive relapse (5). Under these circumstances, different environmental factors may be the cause of separate relapses and further new-onset findings. Therefore, the multiple hit of non-genetic factors may determine the characteristics of full-blown disease.

## Figures and Tables

**Table 1:**
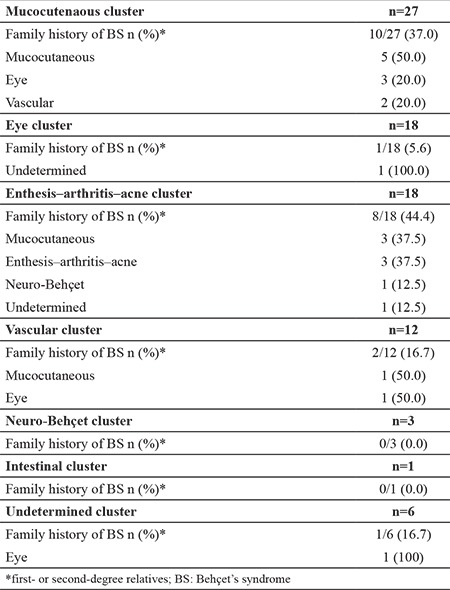
Features of the clusters’ family histories
